# Severe Eosinophilic Asthma

**DOI:** 10.3390/jcm8091375

**Published:** 2019-09-02

**Authors:** Agamemnon Bakakos, Stelios Loukides, Petros Bakakos

**Affiliations:** Department of Respiratory Medicine, National and Kapodistrian University of Athens, 10561 Athens, Greece

**Keywords:** severe asthma, eosinophil, inflammation, interleukin-5 (IL-5), anti-IL-5, interleukin-4

## Abstract

Asthma is a heterogeneous disease with varying severity. Severe asthma is a subject of constant research because it greatly affects patients’ quality of life, and patients with severe asthma experience symptoms, exacerbations, and medication side effects. Eosinophils, although at first considered insignificant, were later specifically associated with features of the ongoing inflammatory process in asthma, particularly in the severe case. In this review, we discuss new insights into the pathogenesis of severe asthma related to eosinophilic inflammation and the pivotal role of cytokines in a spectrum that is usually referred to as “T2-high inflammation” that accounts for almost half of patients with severe asthma. Recent literature is summarized as to the role of eosinophils in asthmatic inflammation, airway remodeling, and airway hypersensitivity. Major advances in the management of severe asthma occurred the past few years due to the new targeted biological therapies. Novel biologics that are already widely used in severe eosinophilic asthma are discussed, focusing on the choice of the right treatment for the right patient. These monoclonal antibodies primarily led to a significant reduction of asthma exacerbations, as well as improvement of lung function and patient quality of life.

## 1. Severe Asthma and Eosinophils

It is well established that asthma is a disease with a great spectrum of symptoms among patients and wide differences in treatment efficacy. In particular, severe asthma is noted to include various specific phenotypes and endotypes, which differ in their clinical presentation, their unique pathogenetic mechanisms, and their responsiveness to treatment [1]. In order to determine the severity of asthma, it is crucial to evaluate patients’ responsiveness to the controller therapy, such as inhaled corticosteroids (ICS) and long-acting β2 agonists (LABA). In other words, clinicians need to evaluate how difficult it is to control asthma symptoms and exacerbations [2]. Therefore, severe asthma presents a challenge as it is defined as a disease which cannot be handled by conventional means of treatment—a medium–high dose of ICS combined with LABA or even oral corticosteroids [3]. A different approach has to be taken to improve asthma outcomes in these patients, and researchers began analyzing the cellular mechanisms that characterize severe asthma. The results were quite intriguing, as they yielded a number of different “types” of severe asthma which have to be recognized and treated accordingly. Eosinophils emerged as the hallmark of a prevalent type of severe asthma, which also involves T cells (T helper 2 (Th2) mainly, but also type 2 innate lymphoid cells) and was labeled the T2-high endotype [4].

Eosinophils were described almost 150 years ago by Paul Ehrlich as granulocytic leucocytes with a bilobed nucleus. Their primary location is within tissue and not in the bone marrow, residing mostly in the gastrointestinal tract in normal conditions [5]. They contain numerous cationic proteins, with four being the most notable: major basic protein (MBP), eosinophil cationic protein (ECP), eosinophil peroxidase (EPO), and eosinophil-derived neurotoxin (EDN); they are mostly associated with parasitic infections, since they have the ability to orchestrate the immune response against helminths in a Th2 cytokine cascade, very similar to that in asthmatic patients with the Th2 endotype [6]. This cascade commences when immunoglobulin E (IgE) reacts with an antigen; although, in helminthic infections, the antigen is indeed threatening for the host, in asthmatic patients, IgE targets are rather innocuous agents such as tree pollen or animal fur. Nevertheless, IgE activates mast cells, macrophages, and basophils, which in turn lead to the production of histamine and other inflammatory cytokines. The ongoing inflammatory process attracts cluster of differentiation 4 (CD4)^+^ T cells and more eosinophils to the site of damage in this aberrant reaction, leading to the Th2-high endotype of severe asthma with high blood and sputum eosinophils [7].

## 2. Eosinophil Production and Development in the Bone Marrow

First of all, it is important to distinguish the two major types of eosinophils in the lungs. Even though the simplistic view we had in the past about eosinophils is still viable, recent studies showed that, except for eosinophils that emerge from the bone marrow and are directly recruited to sites of inflammation, a distinct type of eosinophil with different characteristics resides in tissues in homeostatic conditions. This eosinophil population is called “homeostatic eosinophils” (hEos) [8]. These hEos were mostly examined in mice, and they differ from the regular inflammatory eosinophils (iEos). Both of these populations are produced in the bone marrow from the CD34^+^ progenitor stem cells and specialized CD34^+^ interleukin-5 receptor (IL-5R)^+^ hematopoietic progenitor cells via a complex activation of transcription factors, the most important being GATA-1, PU.1, and CAAT enhancer-binding proteins α and ε [9]. Notably, the actions of GATA-1 and PU.1 are antagonistic regarding the differentiation of other hematopoietic cells; nevertheless, they synergize when it comes to eosinophil production, as it was shown in studies where in vitro enhancement of PU.1 resulted in an amplified GATA-1 transcriptomic effect [10].

Several cytokines also take part in the development of eosinophils apart from the transcription factors previously mentioned, IL-5, IL-3, and GM-CSF(Granulocyte-macrophage colony-stimulating factor). IL-3 and GM-CSF are not selective, and they stimulate the development of other leucocytes such as neutrophils and macrophages more efficiently; however, IL-5 solely affects eosinophils and basophils [11]. The major difference in the production and recruitment of the two eosinophil populations is that hEos are differentiated in the bone marrow semi-independently from IL-5, while iEos need IL-5 in order to be produced from their precursor cells and trafficked to the lungs [12]. This was proven in IL-5 knock-out (KO) mice which could not produce a Th2-high response due to the lack of IL-5, but the number of hEos in the lung was only reduced by half, meaning that they could be recruited via different pathways. It also explains why, in patients treated with an anti-IL-5 agent, eosinophils can still be found in both their blood and lungs [13]. Moreover, stimulation of CD34^+^ progenitor cells with IL-3, IL-5, and GM-CSF resulted in an upregulation of the IL-5R in these stem cells, thus prolonging eosinophil differentiation as long as they were stimulated by IL-5 [14]. However, mice that were lacking GM-CSF/IL-3/IL-5 functions were not observed to have a complete halt of eosinophil production. Instead, these mice had the ability to produce low numbers of eosinophils, indicating that there are more unidentified factors taking part in their development [15].

Even more interesting is the observation that hEos do not take active part in the allergic inflammation, as well as halting this aberrant response. They express several genes that cannot be found in the normal iEos that take part in the immunoregulation of lung and reduce the Th2 response after contact with allergens. Mice who were stripped of the prevalent eosinophil production gene (ΔdblGATA) showed a more severe allergic reaction after contact with dust mites, proving that hEos do not participate in the inflammatory process and they also downregulate the Th2 response, most probably by inhibiting the functionality of dendritic cells [12].

## 3. Eosinophil Migration to the Lung

Eosinophil trafficking from the bone marrow to the lungs is the first major step of the blooming inflammatory process. Even though various chemoattractants were discovered, most of them are not selective and can also draw other leucocytes. Activated Th2 cells and type 2 innate lymphoid cells (ILC2) synthesize IL-4, IL-5, and IL-13, while eotaxin-1 (CCL11) is produced by epithelial and endothelial cells after an allergen challenge. Specifically, IL-5 and eotaxin-1 play a pivotal role in eosinophil trafficking and synergize in promoting lung eosinophilia [16]. Although IL-4 is not a direct eosinophil mediator, it is crucial in the activation of the IgE cascade while also promoting the development of more Th2 lymphocytes, thus sustaining the migratory process [17]. The same applies to IL-13 as it induces eotaxin production [18].

IL-5 is the most crucial cytokine not only in recruiting eosinophils but also in prolonging their survival in tissues. This was observed in IL-5 KO mice which showed a greatly reduced number of eosinophils in the lungs when compared to IL-5 transgenic mice [19,20]. This is largely attributed to the IL-5 receptor that is also expressed in mature eosinophils apart from their progenitors, thus being able to respond to the stimulus of the cytokine via a Janus kinase signal transducer and prolong their half-life (T_1/2_) by almost 50% [21]. IL-5 was also administered routinely to guinea pigs over a period of time, resulting in a reduction of eosinophils in the bone marrow and a concomitant increase of their number in circulation, indicating that it clearly mobilizes them and aids their trafficking into tissues [22]. It is synthesized mostly by activated Th2 lymphocytes and in smaller proportions by eosinophils and mast cells. IL-5 is already the primary target of monoclonal antibody treatment in asthmatic patients, highlighting even more its central role in the pathogenesis of the T2-high inflammatory response. Another source of IL-5 is innate lymphocytes termed ILC-2 cells that may initiate or amplify eosinophilic inflammation. These cells may also produce other Th2-related cytokines such as IL-4, IL-9, and IL-13. That is why the cytokine pattern above is usually termed as T2 instead of Th2 [23].

Eotaxin-1 was first described as a novel component in the bronchoalveolar lavage (BAL) of guinea pigs sensitized and challenged by ovalbumin and was later isolated from human tissue as well. It was the first eosinophil-specific chemoattractant discovered until two more CC chemokines named eotaxin-2 and eotaxin-3 were isolated later on [24]. Eotaxins are produced by epithelial cells of the lung but also in lower numbers by eosinophils, mast cells, macrophages of the alveoli, vascular endothelial cells, and smooth muscle cells of the airways after stimulation by IL-4 and IL-13 [25]. Eosinophils express receptors for the CC groups of chemokines, a classic G-protein transmembrane receptor, while eotaxins interact specifically with the CCR3 receptor and synergize with IL-5 and between themselves, in order to recruit eosinophils to the lungs [26]. It should be noted that the CCR3 receptor is constantly expressed on the eosinophil membrane, but its expression is further increased after an inflammatory stimulus [27]. Characteristically, it was demonstrated that the airways of asthmatic patients have a higher number of cells producing messenger RNA (mRNA) for CCR3 and its ligands, compared to healthy individuals [28]. Other cells expressing CCR3 receptors are basophils, mast cells, Th2 cells, and eosinophil progenitor cells. Activation of the CCR3 receptor by eotaxin results in the internalization of the ligand and induces chemotaxis via calcium mobilization and actin polymerization [29]. Studies showed that all three eotaxins are upregulated after an allergen challenge and have a pivotal role in different phases of the immune response.

Eotaxin-1 is needed in the first steps of the inflammatory response, whereas eotaxins 2 and 3 are needed to prolong eosinophil survival later on [30]. Their synergistic role is clearly demonstrated in studies between single eotaxin-1 or eotaxin-2 KO mice and both eotaxin 1 and 2 KO mice. The latter group had far fewer eosinophils in their lungs after an allergen challenge when compared to the single KO group [31]. Eotaxin-1 is also crucial in mobilizing eosinophils from the bone marrow, with its levels being correlated with the eosinophil number in blood and lungs in pig specimens. However, inhibition of IL-5 in those pigs showed that eotaxin-1 alone cannot mobilize eosinophils from the bone marrow, thus highlighting the importance of the cooperation between those two chemokines [32]. The same applies to their mobilization from circulation to tissues, since administration of eotaxin-1 without abolishing IL-5 effects increases blood eosinophilia but fails to increase their number in tissue. On the contrary, administration of IL-5 without abolishing eotaxin-1 demonstrated a notably higher number of tissue eosinophils, further underlining the important role of IL-5 in the tissue infiltration process [33]. Eotaxin-2 synergizes with IL-5 and drives the production of IL-13, with which it later synergizes to promote lung eosinophilia [34]. Eotaxin-3 levels start to rise at a later time, and it is thought to prolong the eosinophil recruitment in lungs [35]. The CCR3 receptor was targeted for the development of new targeted treatment of asthma, since it is expressed in all the cells taking part in the inflammatory process, making it evident that blocking the eotaxin/CCR3 axis might prove greatly important in future trials.

Eosinophils, which are found in circulation after being mobilized mostly by IL-5 and eotaxin-1 as previously mentioned, still need to migrate from the vasculature to the lung tissue. In this phase, several eosinophil-specific adhesion molecules with the most important being the β1 integrin very late antigen (VLA-4), the vascular cell adhesion molecule (VCAM-1), and the P-selectin glycoprotein ligand (PSGL-1) play an important role [36]. VLA-4 is an integrin which is expressed on the membrane of eosinophils after a stimulus from eotaxin-1. It ligands with the VCAM-1 integrin expressed at the vasculature membrane, resulting in the activation and firm adhesion of eosinophils to it, aiding their transit from the endothelium to tissues [36]. Usage of inhibitors of the VLA-4 and VCAM-1 interaction in mice studies showed a greatly reduced inflammatory response and eosinophil number in lungs, compared to normal mice [37]. Apparently, this ligand is a selective eosinophil adhesion chemokine, since it does not cause the adhesion of other leucocytes to the endothelium; therefore, more research is needed as to whether a VLA-4/VCAM-1 inhibitor could be used in the treatment of severe eosinophilic asthma. PSGL-1 on the other hand binds to P-selectin and modulates the first steps of the interaction between eosinophils and the endothelium, more specifically the rolling and adhesion stages. It is also solely expressed by eosinophils, which means that tampering with the PSGL-1/P-selectin ligand can reduce the transit of eosinophils to tissue. Trials were conducted in mice with ablation of the P-selectin gene and, indeed, those mice had fewer eosinophils in their lungs [38]. Inhibitors targeting this selectin ligand are currently being investigated in clinical trials; however, they are yet to yield promising results.

Recent studies highlighted the fact that eosinophilopoiesis can also occur in situ in the airways of severe asthmatics, since the number of CD34^+^ and CD34^+^ IL-5Rα^+^ hematopoietic progenitor cells was much higher in this population’s sputum when compared to mild asthmatics. Even more interesting was the fact that eosinophil progenitor cells did not vanish after anti-IL-5 treatment in these patients, which means that in situ eosinophilopoiesis is an important mechanism of persistent eosinophilia in the airways [39]. This could be attributed to the action of bronchial epithelial cells which, after being triggered by an extraneous stimulus, produce several cytokines, such as IL-25 and IL-33, along with thymic stromal lymphoid proteins (TSLPs) known as alarmins. Their expression was found to be higher in the airways of asthmatic patients, while they also correlate with disease severity [40]. The T2 cascade is sustained by the production of these alarmins, since they can promote eosinophil progenitor cell recruitment and trigger T2 cells, especially ILC2 cells, in producing IL-4, IL-5, and IL-13 [41]. This persistent production of cytokines by ILC2 cells facilitates the eosinophil progenitor cell homing to the lungs and provides fertile soil for their in situ maturation, causing persistent eosinophilia in these patients [42].

## 4. Eosinophilic Inflammation in the Lung

Eosinophils are the predominant cells of the inflammatory response in the lungs, contributing greatly to two major events: the remodeling and the hyperresponsiveness of the airways (AHR). Persistent inflammation caused by eosinophils leads to constant damage of the airways. The regeneration process is not flawless and results in hypertrophy of the smooth muscles, hyperplasia of goblet cells, and deposition of extracellular matrix proteins, causing membrane thickening and fibrosis [43].

The damage caused at the bronchial level is attributed to the degranulation of eosinophils and the release of their toxic proteins. Degranulation can occur in three different ways: (i) exocytosis, (ii) piecemeal degranulation, and (iii) cytolysis. In exocytosis, most specifically the subtype compound exocytosis, multiple granules inside the cell fuse and are then secreted to the extracellular space. This is the classic way that eosinophils act against helminths [44]. Piecemeal degranulation was demonstrated to be the most prevalent mechanism of eosinophil degranulation in asthmatic patients. In this highly regulated mechanism, the cytoplasmic proteins are “packaged” selectively in small vesicles, and then transported to the membrane through a tubulovesicular system until they are finally released by exocytosis. Various chemokines carefully regulate this process, such as Interferon-gamma (IFN-γ) and eotaxin-1, with studies showing that stimulating human eosinophils with a cytokine leads to the selective release of an eosinophilic protein [45,46]. In cytolysis, the cell dies; however, unlike apoptosis, its granules are released in the microenvironment, fully potent and active. Eosinophils which do not undergo piecemeal degranulation release their content through cytolysis [47]. Even if these mechanisms normally exist to protect tissues from damage, in this inflammatory process, the released proteins damage the epithelium, increase vascular permeability, and activate mast cells [48].

The release of eosinophilic granules and other mediators was proven to damage the airways in multiple ways. The smooth muscles of the airways contract via the M3 receptor after being triggered by acetylcholine. The M2 receptor limits its release and acts as a regulatory mechanism [49]. Eosinophils release MBP which is an allosteric antagonist of the M2 receptor, leading to an uncontrollable stimulation of the M3 receptor by acetylcholine and, thus, to bronchoconstriction [50]. MBP and other eosinophilic proteins were also shown to damage epithelial cells in vitro in similar concentrations to those found in the lungs of asthmatic patients, further proving their toxic effects [51]. However, MBP-abolished mice were not protected from AHR, meaning that other factors also contribute to this process [52]. Leukotrienes are abundant inside eosinophils, and their release causes bronchoconstriction and activates mast cells and basophils, which also excrete prostaglandins, histamine, and more leukotrienes to support the ongoing inflammation [53]. Eosinophils may induce AHR in a more indirect way, since eosinophil-ablated mice could still develop AHR when injected with T cells producing IL-13, which was demonstrated to cause AHR despite the absence of eosinophils [54]. More studies highlighted this indirect effect on AHR, since mast cells were proven to be more important in developing AHR in patients with eosinophilic asthma [55]. Blocking both CCR3 and IL-5 experimentally could not distinguish the effects of eosinophils and mast cells in AHR, since CCR3 is expressed in both types of cells. Nevertheless, use of CCR3 antagonists showed a significant reduction of both AHR and airway remodeling in animal studies, demonstrating the importance of the CCR3/eotaxin-1 axis [56]. Genetic ablation of eosinophils in mice via the *GATA-1* gene showed no protection from AHR when compared to normal mice in asthmatic models [57]. Therefore, while AHR is definitely one of the hallmarks of asthma, its correlation with eosinophils is debatable and seems to be more of a secondary effect of the generalized inflammatory process.

Nevertheless, eosinophils were proven to be one of the main factors behind airway remodeling. In a study designed with the same concept as the previous one mentioned, Δdbl-GATA mice were challenged by allergens and compared with wild-type mice. The latter group was found to exhibit all the features of airway remodeling, whereas the eosinophil-naïve mice were protected from it [58]. Similar results were demonstrated in both IL-5 KO mice and patients treated with anti-IL-5 agents, proving that reducing the number of eosinophils indeed reduces the deposition of extracellular matrix proteins (ECMs) such as collagen I in the airway lumen [59,60,61]. Eosinophils are activated by the effect of tumor necrosis factor-alpha (TNF-α) and, as recent studies showed, by IL-1beta; they secrete matrix metalloproteinase-9 which is one of the main enzymes found in asthmatic patients, highly correlated with the remodeling process and the persistent recruitment of eosinophils [62,63]. They also are a potent resource of transforming growth factor-β (TGF-β) which acts as a chemoattractant for fibroblasts and activates local fibroblasts to differentiate into myofibroblasts and even into smooth muscle cells, inducing ECM production in the meantime [64]. Mice treated with an anti TGF-β agent did not show evidence of airway remodeling, even if the inflammatory process was not altered, highlighting the pivotal role of TGF-β—mostly its correlation with the thickening of the basement membranes [65]. TGF-β is not only an eosinophil product; its mRNA was found increased in all the inflammation stages, with reports suggesting that eosinophils are its primary source in the first stages of the disease [61]. Nitric oxide (NO) is another toxic molecule secreted from eosinophils, and its levels correlate with the biomarker FeNO which is discussed later on [66]. Reactive oxygen species (ROS) are yet another product of eosinophils with clear potential to damage the airway and induce a fibrotic process [67]. Summarizing, eosinophils clearly contribute to airway remodeling, and the inhibition of eosinophil adhesion and activation may also reduce the inflammatory process and airway remodeling.

## 5. Biomarkers in Severe Eosinophilic Asthma and Endotyping

There was always a notion that the heterogeneity of asthma is due to the different phenotypes and endotypes of the disease. Nevertheless, endotyping became a necessity throughout the years; therefore, the need for specific biomarkers of every distinct type increased. These biomarkers include serum IgE, blood eosinophil levels, sputum eosinophils, and levels of exhaled nitric oxide in breath (widely known as FeNO) [68].

Sputum eosinophils are the most interesting biomarker in severe eosinophilic asthma due to the insight they provide into airway eosinophilia, despite the difficulty of collecting and analyzing them in every patient routinely. Treatment of patients based on sputum eosinophils showed a reduction of the rate of exacerbations, especially in those with severe asthma. [69] Both European Respiratory Society/American Thoracic Society (ERS/ATS) and Global Initiative for Asthma (GINA) guidelines support the use of sputum eosinophils for severe asthma management [1]. Sputum eosinophils ≥ 3% are correlated with airway eosinophilia [70]. Sputum mRNA can also be used in order to determine whether patients belong in the T2-high or the T2-low group, according to the expression of cytokines found in their sputum. Although this is a more costly method, it can “mark” candidates for biological treatments [71].

Blood eosinophils were used in the past few years as a marker for severe eosinophilic asthma requiring biological treatment with an anti-IL-5 agent, since they are correlated with sputum eosinophils. The threshold was put in several counts during trials, with the most often picked numbers being 150 cells/μL or 300 cells/μL; however, the most important from a clinical point of view is that blood eosinophil count—an easy and inexpensive biomarker—was chosen over sputum eosinophil number for eligibility for anti-IL-5 therapy [72]. During the anti-IL-5 trials, many biomarkers were evaluated, but none were deemed superior to blood eosinophils. The use of blood eosinophil counts as a biomarker for airway eosinophilia is based upon the relationship between blood and sputum eosinophil counts [73]. However, it should be noted that, although airway eosinophils are considered to better reflect eosinophil involvement in airway inflammation, peripheral blood eosinophils do not necessarily parallel airway eosinophils. High blood eosinophil numbers present good specificity for airway eosinophilia [74,75]. On the other hand, low blood eosinophil numbers might not accurately reflect the absence of airway eosinophilia [76,77]. This was demonstrated in a study including children with severe asthma, in which, despite 86% of them having blood eosinophil counts within normal levels, 84% still presented airway eosinophilia [78]. It should also be taken under consideration that blood eosinophil counts are influenced by high-dose ICS and mainly oral corticosteroids (OCS) [79]. A single measurement of blood eosinophil count of at least 150 cells/μL was shown to predict subsequent measurements on average of at least 150 cells/μL in 85% of patients [80].

FeNO is another marker that is used commonly and can inform us about the ICS response we should expect from a patient [81]. Nevertheless, there are several protruding factors that can confuse the results, the most important being smoking, allergic rhinitis, and female gender [82,83]. FeNO >50 ppb in adults suggests the presence of Th2-high inflammation, whereas FeNO < 25ppb suggests a Th2-low process. In another study, it was shown that, in patients with severe asthma refractory to treatment, an FeNO level >19ppb was indicative of sputum eosinophilia [84]. However, current guidelines from ATS/ERS do not recommend FeNO-guided management for patients with severe asthma [1]. This could be attributed to the fact that FeNO is correlated with the NO produced in asthmatic airways by other cells apart from eosinophils, such as epithelial cells and macrophages. Thus, NO cannot be solely linked to eosinophils and the need for biological treatment, and it is more likely correlated with other aspects of the Th2 inflammation [85].

Volatile organic compounds (known as VOCs) are a modern biomarker also found in exhaled breath, like FeNO, and they are bound to predict with great accuracy both eosinophil and neutrophil counts in blood, while they are also correlated with eosinophil number in BAL [86]. They are processed by a meticulous algorithm called eNOSE (electronic nose), and early research suggested that they could be superior in estimating the risk of exacerbations and insensitivity to corticosteroids [87]. A recent study demonstrated that particular VOCs (hexane and 2-hexanone) had a high classification performance for eosinophilic asthma in a large asthmatic population classified according to their sputum cell count. Moreover, the combination of FeNO, blood eosinophils, and VOCs gave a very satisfactory prediction of eosinophilic asthma with an area under the curve (AUC) of 0.9 [88]. However, more data are needed if this method is to be applied on a daily basis. Last but not least, serum periostin, which derives from epithelial cells of the lung after stimulation by IL-13, was used as a biomarker of the T2-high endotype [89]. It was used in various studies as a predictor of Th2 inflammation and, even though the BOBCAT study showed that it was superior to regular biomarkers, the follow-up studies could not support these findings [75].

A combination of biomarkers may be better than using one alone, and this trend was followed in many studies. In the U-BIOPRED cohort study, a specific endotype of severe asthma involving eosinophils was described as “late-onset asthma with past or current smoking and chronic airflow obstruction with a high blood eosinophil count” [90]. A similar endotype was discovered by both the SARP and the Leicester cohorts using blood eosinophilia as an inflammatory marker, describing “late onset asthma associated with nasal polyps and resistance to corticosteroid therapy” and “a late-onset disease along with rhinosinusitis and numerous exacerbations”, respectively [91]. The majority of these patients needed oral corticosteroids to achieve control of the disease and minimize exacerbations [92]. Although endotyping may not seem simple, it reveals individual therapeutic targets by means of specific treatable traits and mechanisms, leading to precision medicine, with the aid of biomarkers. For instance, Th2-high patients with severe asthma under ICS and LABA had higher FeNO, as well as blood and sputum eosinophil counts, compared to those with Th2-low inflammation in research using the *IL-13* genes in epithelial cells of the bronchial tree.

Concluding, it is clear that biomarkers have a role to play in guiding therapy of severe asthma. However, a combination of biomarkers may be used in order to achieve a greater predictive value. Also, new biomarkers with better correlation to specific endotypes and their respective molecular pathways need to be discovered in order to achieve optimal therapy.

## 6. Anti-IL-5 Therapy in Severe Eosinophilic Asthma

### 6.1. Mepolizumab

The story of anti-IL-5 treatment in asthma is definitely a fascinating one. Given the central role of eosinophils both in the allergic and non-allergic cascade of asthmatic inflammation, along with the fact that IL-5 is the cytokine mainly responsible for the differentiation, maturation, airway trafficking, and survival of eosinophils, the development of monoclonal antibodies against IL-5 raised high expectations for new treatment approaches, primarily in severe asthma.

However, the first studies were somewhat disappointing. In one study, mepolizumab prevented the rise in eosinophil numbers both in blood and sputum after inhaled allergen challenge, but it did not ameliorate allergen-induced asthmatic responses [93]. In another study including a small number of patients with difficult-to-treat asthma who were receiving high-dose ICS and/or oral CS, anti-IL-5 was able to reduce blood eosinophils but did not have an effect on other clinical outcomes apart from a small improvement in lung function— forced expiratory volume in the 1st second (FEV1) [94]. A few years later, in another study, mepolizumab was administered in a large group of not well-controlled patients with moderate to severe asthma, despite being treated with ICS and receiving four puffs of beta2-agonist daily as recue medication. Again, anti-IL-5 diminished blood eosinophils but did not manage to improve any clinically important outcome [70]. In the high-dose group, there was a trend toward reducing severe exacerbations, but the study was not powered to show such an effect. In spite of the consistent effect of anti-IL-5 in the reduction of blood eosinophils, the lack of a favorable effect in clinical asthma outcomes was obvious. These findings supported the dismal statement of the “final nail in the coffin for anti-IL-5 treatment in asthma”.

However, in 2009, two small but well-designed randomized controlled trials were contracted that meant to change the road of anti-IL-5 treatment in asthma. In the first study, 20 asthmatics received either mepolizumab or placebo at five monthly intravenous infusions. These patients had corticosteroid-resistant eosinophilic asthma, and it is important to note that, although they were receiving a median dose of 10 mg of prednisone for a mean time of nine years and a high ICS dose, they still had >10% sputum eosinophils [95]. In the second study, 61 asthmatics received 12 infusions of either mepolizumab or placebo monthly [85]. Both studies revealed a significant reduction of exacerbations, accompanying a significant reduction in blood and sputum eosinophils. In the first study, the reduction of exacerbations occurred along with a reduction in prednisone dose. Still, there was no other clinically meaningful improvement in symptoms or lung function (FEV1) in both studies. These studies highlighted the importance of eosinophils in the pathogenesis of asthma exacerbations, but more clearly paved the way for the future of anti-IL-5 treatment by focusing—in contrast to previous studies—on two main determinants. Firstly, the primary outcome benefit from anti-IL-5 treatment relies mainly on the reduction of exacerbations; secondly, this benefit is obvious when selecting asthmatics with persistent eosinophilic inflammation despite regular corticosteroid (inhaled and/or oral) treatment.

Apart from a clear link with exacerbations, eosinophils are also important in airway remodeling in asthma. TGF-beta derived from eosinophils is involved in this process. In a study including 24 atopic asthmatics, anti-IL-5 treatment with mepolizumab reduced airway eosinophil numbers and significantly decreased the expression of three extracellular matrix proteins (tenascin, lumican, procollagen III) in the reticular basement membrane. It also reduced the percentage and the number of eosinophils expressing TGF-beta 1. These findings are extremely important, especially taking into consideration that the asthmatics included in this study were mild and received only short acting beta agonists (SABA) and not ICS. Firstly, these findings indicate that remodeling is present even in mild asthma, and it is driven to some degree by eosinophil-derived TGF-beta 1; secondly, anti-IL-5 can prevent this process by regulating the TGF-beta-enhanced deposition of matrix proteins through the reduction of eosinophils [60].

One of the largest studies in severe asthma, the DREAM study, including 621 patients was undertaken in order to examine the effect of mepolizumab in reducing the rate of clinically significant exacerbations. As such were defined the exacerbations that required oral corticosteroids or visit to an emergency department or hospitalization. All asthmatics had a history of at least two exacerbations requiring systemic corticosteroids in the previous year and signs of eosinophilic inflammation despite treatment. These signs were either sputum eosinophils >3%, peripheral blood eosinophils > 300 × 10^6^/L, FeNO > 50 ppb, or loss of asthma control after a ≤ 25% reduction in regular corticosteroid dose (inhaled or oral). The study had a duration of 52 weeks, and patients received 13 infusions of one of three doses of IV mepolizumab (75, 250, and 750 mg). All three doses equally and significantly reduced the rate of asthma exacerbations. Moreover, they reduced the number of blood and sputum eosinophils and they were well tolerated. No improvements in FEV1 and AQLQ (asthma quality of life questionnaire) were observed, and this, in accordance with previous studies, indicated the dissociation of measures of control and exacerbations. This study also provided clinically valuable information regarding predictors of efficacy of mepolizumab treatment. The two main determinants were the baseline peripheral blood eosinophil number and the number of exacerbations in the previous year. Higher numbers indicated a more likely response to treatment. Other factors such as baseline FEV1, acute response to bronchodilators, IgE level, and atopic status were not associated with probability of response to mepolizumab [73].

In a following study (MENSA), 576 asthmatics treated with high-dose ICS with or without oral corticosteroids were randomized to receive either 75 mg of mepolizumab IV, 100 mg of mepolizumab subcutaneously (SC), or placebo every four weeks for 52 weeks. These asthmatics had at least two exacerbations requiring systemic corticosteroids the previous year and evidence of eosinophilic inflammation reflected by an eosinophil count of 150 cells/μL at screening or above 300 cells/μL at some time point in the previous year. The primary outcome was the annualized rate of exacerbations, and they were significantly reduced by both IV and SC mepolizumab by 47% and 53%, respectively. This was the first study to show that mepolizumab was associated with a significant improvement in lung function (FEV1), quality of life (AQLQ), and asthma control (ACQ-5) [96].

Another study (SIRIUS) explored the systemic corticosteroid-sparing effect of mepolizumab. In total, 135 asthmatics with severe eosinophilic asthma were randomized to receive either mepolizumab (100 mg SC) or placebo every four weeks for 20 weeks, and the primary outcome was the percentage reduction of the oral corticosteroid dose. The evidence of eosinophilic asthma was determined—similar to MENSA—by an eosinophil count of 150 cells/μL at screening or above 300 cells/μL at some time point in the previous year. In contrast to the MENSA study where 25% of asthmatics received oral steroids, in SIRIUS, all of the included patients received a mean dose of 10 mg of prednisone. This study involved a so-called optimization phase, in which a reduction of the dose of oral steroids was attempted before the start of mepolizumab, so as to establish that the patients genuinely needed this dose for their asthma control. The study showed that mepolizumab permitted the reduction of oral corticosteroid dose; moreover, despite this reduction, it also significantly reduced the rate of exacerbations and improved asthma control and quality of life (secondary outcomes in this study) [97].

In a 12-month open-label extension study of MENSA after the cessation of mepolizumab treatment, it was found that eosinophils increased both in blood and sputum, returning to pre-treatment levels within three months of cessation. As for asthma control, 12 months after the stop of medication, the exacerbation rates were similar to the pretreatment levels [98]. This study showed deterioration in exacerbation frequency after the cessation of mepolizumab that was preceded by a rebound worsening of eosinophilic inflammation.

In a post hoc analysis of the DREAM and MENSA studies, patients were stratified according to baseline blood eosinophil count in order to evaluate whether this biomarker could be used to predict response to mepolizumab. It was shown that using a threshold of 150 cells/μL could predict a favorable outcome in reducing exacerbations. Most importantly, this reduction was higher with increasing baseline blood eosinophil count (52% versus placebo for those with baseline blood eosinophils >150 and 70% for those with baseline blood eosinophils >500 cells/μL) [99]. In a subgroup analysis of the studies DREAM, MENSA, SIRIUS, and MUSCA, it was demonstrated that asthmatics with baseline eosinophils 150–300 cells/μL showed benefits in terms of reducing exacerbations and reducing the need for systemic corticosteroids that were clinically meaningful and comparable to patients with baseline >300 eosinophils/μL [100].

In patients with severe eosinophilic asthma previously treated with omalizumab, a post hoc analysis from MENSA and SIRIUS demonstrated that the response to mepolizumab was the same regardless of previous use of omalizumab [101]. This is clinically important because a subgroup of patients eligible for mepolizumab is also eligible for omalizumab treatment. Accordingly, a lack of response to omalizumab does not preclude a favorable response to mepolizumab in such asthmatics.

Another 32-week study (OSMO) included 145 patients who were eligible for both omalizumab and mepolizumab and were not controlled with omalizumab (median time of omalizumab treatment was 29.6 months). These asthmatics were switched immediately after the last dose of omalizumab to mepolizumab and achieved significant improvement, reflected by a 64% reduction in exacerbations compared to the previous year, better asthma control (measured by ACQ-5), and better quality of life (measured by Saint George’s respiratory questionnaire—SGRQ). These outcomes were achieved early within 8–12 weeks and were kept or even improved during the study, indicating no evidence of possible additional action of the two antibodies until the wash-out of omalizumab. This study provided support to clinical practice in terms of switching from one biologic agent to another [102].

A study (MUSCA) assessed the effect of mepolizumab in the quality of life of patients with severe eosinophilic asthma and found a significant improvement in SGRQ of 7.7 (surpassing the minimal clinically important difference of four units), with a safety profile comparable to placebo [103].

Regarding safety, in a 52-week, open-label extension study of MENSA and SIRIUS (COSMOS study), mepolizumab had a favorable long-term safety profile, without any increase in the rate of adverse events [104]. Similarly, in the COLOMBUS study, an extension of the DREAM study lasting 3.5 years with a maximum exposure of 4.5 years, mepolizumab was safe and maintained its efficacy in the reduction of exacerbations [105].

Using data from five phase III studies with mepolizumab, it was shown that few patients developed anti-drug antibodies that had no impact on safety or efficacy of mepolizumab. Only one patient (from the SIRIUS study) was positive for neutralizing antibodies, but pharmacokinetic samples were not quantifiable during follow-up. These data show the low immunogenic response of mepolizumab [106].

### 6.2. Reslizumab

Reslizumab is a humanized anti-IL-5 IgG4 monoclonal antibody that binds with high affinity to the alpha subunit of the cytokine IL-5, thus preventing the interaction with its receptor [107].

Initially, a pilot safety study including 32 asthmatics showed that reslizumab at a dose of 1 mg/kg given intravenously reduced blood and sputum eosinophils but had no effect in lung function and airway hyperresponsiveness [94]. In the following phase IIb randomized, double-blind, placebo-controlled study, 106 patients with asthma and sputum eosinophils ≥3% were administered reslizumab at a dose 3 mg/kg IV every four weeks. Reslizumab managed to decrease sputum eosinophils significantly and improve FEV1, as well as improve asthma control (ACQ) in those patients with nasal polyps [108].

The two main phase III studies included 953 asthmatics that were randomized to receive either reslizumab (3 mg/kg IV) or placebo. All included patients had a baseline peripheral blood eosinophil count of > 400 cells/μL, ACQ-7 >1.5, at least 12% FEV1 reversibility, and at least one exacerbation requiring OCS in the last year; they were also on regular treatment with high-dose ICS plus additional controller with or without OCS (up to 10 mg of prednisone). The duration of the studies was 52 weeks, and the primary outcome was the rate of exacerbations defined either as need for OCS or doubling the ICS dose. Reslizumab was effective in reducing exacerbations significantly, improving FEV1, ACQ-7, and AQLQ, as well as reducing rescue medication and blood eosinophils [109]. In a post hoc analysis of these two studies, it was demonstrated that late-onset asthma (defined as onset after the age of 40) showed a better response to reslizumab compared to early-onset asthma [110].

In conclusion, these studies showed that reslizumab at a dose of 3 mg/kg IV is safe and more effective in patients with severe eosinophilic asthma and a peripheral blood eosinophil count > 400 cells/μL.

In another study including 10 patients with oral corticosteroid-dependent asthma, weight-adjusted intravenous reslizumab was more effective in reducing sputum eosinophilia compared to fixed-dose SC mepolizumab that was administered for at least one year with inadequate response. This was associated with a greater improvement in asthma control measured by ACQ-5 [111].

### 6.3. Benralizumab

Benralizumab is a humanized, afucosylated, monoclonal antibody targeting the IL-5α receptor. In comparison to anti-IL-5 monoclonal antibodies, benralizumab induces a direct, fast, and nearly complete depletion of blood eosinophils through enhanced antibody-dependent cell-mediated cytotoxicity, via natural killer cells [112]. As IL-5 receptors are expressed not only on eosinophils, but also on eosinophil progenitors and basophils, it is expected to affect all these populations. A study evaluating the effect of benralizumab on eosinophils in different compartments such as bone marrow, peripheral blood, sputum, and airways showed that bone marrow and peripheral blood eosinophils were completely suppressed while airway eosinophils (tissue and sputum) were also extensively depleted [113]. Two phase III trials, SIROCCO and CALIMA demonstrated that benralizumab significantly reduced the rate of asthma exacerbations in patients with severe, uncontrolled asthma and blood eosinophil counts ≥300 cells/μL [114,115]. In the SIROCCO study, benralizumab administered either every four weeks or every eight weeks (after the first three doses given every four weeks) reduced the rate of exacerbations by up to 51% after 48 weeks of treatment. It also improved lung function (expressed as an increase in pre-bronchodilator FEV1) and asthma control [114]. The effect compared to placebo was greater for the eight-weekly dosage, with the potential to lower the burden of asthma and reduce costs in comparison to other biologics that need to be given on a monthly basis. In CALIMA, a study of similar design to SIROCCO with a duration of 56 weeks, it was confirmed that benralizumab reduced asthma exacerbations up to 36% in patients with severe eosinophilic asthma and blood eosinophil counts ≥ 300 cells/μL. Again, as found in SIROCCO, a substantial improvement in lung function and asthma symptoms was observed [115]. Although there were no direct comparisons between biologics, it seems that the increases in lung function were greater with benralizumab than with other biologics.

In both studies, benralizumab produced a direct, rapid, and nearly complete depletion of eosinophils as early as four weeks, providing support for its mechanism of action directly on the IL-5α receptor, causing eosinophil apoptosis. Benralizumab depletes eosinophils directly, whereas mepolizumab and reslizumab reduce eosinophil number rather than deplete them entirely. This way, benralizumab is likely to overtake potential issues such as the induction of increased cytokine production due to cytokine-directed antibodies. A pooled analysis from the SIROCCO and CALIMA studies demonstrated that benralizumab was safe and effective in patients with severe eosinophilic asthma and blood eosinophils > 150 cells/μL [116].

A subsequent pooled analysis of the SIROCCO and CALIMA studies stratified patients according to baseline blood eosinophil count and by number of exacerbations (two and three or more). In this analysis, the rates of asthma exacerbations were increasingly reduced with increasing blood eosinophil thresholds and with greater exacerbation history. These reductions were even greater with a combination of high blood eosinophils and a history of more frequent exacerbations [117].

Another phase III study, ZONDA, showed that benralizumab significantly reduced the dose of oral prednisone in OCS-dependent patients, while also reducing the rate of exacerbations. All patients received oral corticosteroids for at least six months prior entering the study. The study included a run-in phase where the dose of prednisone was reduced to the minimum while maintaining asthma control, and this preceded the first administration of benralizumab. After 28 weeks, 50% of the patients managed to stop oral corticosteroids, while the likelihood of reducing the dose was four times higher in benralizumab-treated than in placebo-treated asthmatics [118].

A phase III extension study, BORA, included patients who completed the SIROCCO and CALIMA studies and evaluated the safety and tolerability of benralizumab. Interestingly, patients that received placebo in SIROCCO and CALIMA were randomized to receive benralizumab either every four weeks or every eight weeks (after administration of the first three doses every four weeks). The study confirmed the two-year safety of benralizumab as the percentage of patients experiencing adverse events was not different between BORA and the SIROCCO and CALIMA studies. No increased risk of infection was observed in patients receiving benralizumab for two years despite the long-term depletion of eosinophils. Moreover, asthmatics who were treated with benralizumab in BORA but received placebo in SIROCCO and CALIMA showed a comparable reduction in exacerbation rate with those receiving the active drug from the first year [119].

It is of high clinical importance to assess baseline characteristics in patients with severe eosinophilic asthma that may predict the response to treatment with benralizumab. In a study including patients from the SIROCCO and CALIMA phase III studies, it was shown that OCS use, nasal polyps, forced vital capacity (FVC) < 65% pred adult onset of asthma (>18 years), and three or more exacerbations in the previous year were associated with a greater response to benrlizumab, measured either as annual exacerbation rate or change in pre-bronchodilator FEV1 for those with > 300 eosinophils/μL. Interestingly, OCS use, nasal polyps, and FVC < 65% pred could predict a better response to benralizumab in decreasing the rate of exacerbations, even in patients with < 300 eosinophils/μL [120]. This study highlights the importance of assessing these clinical features when evaluating an asthmatic patient eligible for benralizumab, and adds to the already known baseline blood eosinophil count predictive information for responsiveness to treatment.

Another study assessed the effect of benralizumab treatment by stratifying patients according to atopic status (atopic or non-atopic) and IgE level (high > 150 kU/L or low < 150 kU/L). The study again included patients from the phase III SIROCCO and CALIMA studies and demonstrated that the efficacy of benralizumab in reducing the exacerbation rate and improving lung function was not affected by atopic status and serum IgE level [121]. This is clinically important because it indicates that benralizumab is effective in patients with severe eosinophilic asthma that might be eligible for omalizumab treatment as well.

There are no head-to-head trials for direct comparison between the different anti-IL-5 biologics. In a matching-adjusted indirect comparison, benralizumab and mepolizumab similarly reduced exacerbation rate and improved lung function. No comparison could be made between benralizumab and reslizumab due to differences in study populations [122]. Another indirect comparison demonstrated that, in patients with similar blood eosinophil counts, mepolizumab was more effective in reducing exacerbations than benralizumab and reslizumab. As for lung function, benralizumab was associated with a greater improvement in FEV1 compared to reslizumab for patients with a blood eosinophil count > 400 cells/μL [123]. However, all these findings of the indirect comparison trials should be viewed with caution because of the differences in study populations and in the number of exacerbations in the previous year of the included patients. Moreover, there were differences in the treatment the patients received before starting the biologic (either the ICS dose and/or OCS dose). The studies for reslizumab enrolled asthmatics with baseline blood eosinophils > 400 cells/μL, a higher number compared to those enrolled in studies for mepolizumab and benralizumab. Accordingly, a greater effect might have been expected.

There are no studies evaluating possible co-administration of biologics with different mechanisms such as anti-IgE and anti-IL-5 for those who present a mixed phenotype (severe allergic and eosinophilic asthma). In these patients, it is logical to assess the predominant characteristics and decide which biologic to start [124].

It is suggested that anti-IL-5 antibodies be administered for at least 16 weeks in order to assess efficacy. However, this time may be extended up to 12 months as there are some late-responders, and 16 weeks is possibly too short a length of time to evaluate reduction in exacerbations [125].

There are still some unanswered questions with major clinical importance. How long should an anti-IL-5 be prescribed in a patient with severe eosinophilic asthma? It seems that, after stopping it, there is a relapse of exacerbations following an increase in blood and sputum eosinophils, and this was shown with mepolizumab and benralizumab. This relapse of eosinophilic inflammation is compatible with a lack of long-term bone marrow suppression after discontinuation of medication [98,119]. Is there a rationale for moving from an anti-IL-5 antibody to an anti-IL-5 receptor antibody or vice versa? The mechanism of action is different, and benralizumab is associated with almost a depletion of blood eosinophils while mepolizumab reduces them significantly but does not deplete them. However, no difference in efficacy in any outcome (exacerbation rate, lung function) was observed. One possible explanation is that these antibodies exert their effect by reducing the eosinophil pool in the bone marrow, thus reducing exacerbations through the reduction of eosinophils that are available for mobilization and trafficking in the airways.

## 7. Anti-IL-4 therapy in Severe Asthma

### Dupilumab

Dupilumab is a fully human anti-interleukin-4α receptor monoclonal antibody, recently approved for moderate-to-severe eosinophilic asthma or oral steroid-dependent asthma. It blocks interleukin-4 and interleukin-13, which are key mediators in type-2-mediated inflammation.

The first study on dupilumab included 52 asthmatics with severe eosinophilic asthma and a baseline blood eosinophil count of > 300 cells/μL or sputum eosinophils > 3% who were treated with medium-to-high-dose ICS plus LABA. The patients received dupilumab (300 mg SC) or placebo weekly for 12 weeks or until an exacerbation occurred. The design of the study was provocative since asthmatics discontinued LABA by week four and gradually tapered and discontinued ICS at weeks 6–9. Dupilumab was associated with an 87% reduction in exacerbations compared to placebo and also improved lung function and reduced markers of Th2 inflammation [126].

The following phase IIb study included 769 patients with severe asthma on medium-to-high ICS plus LABA, irrespective of baseline blood eosinophil count. They received 200 mg or 300 mg of dupilumab or placebo every two or every four weeks for a total duration of 24 weeks. Dupilumab improved FEV1 and reduced the exacerbation rate significantly in the total population and also in the subgroups of patients with less than or more than 300 eosinophils/μL [127]. In a post hoc analysis of this study, the favorable effects of dupilumab were demonstrated regardless of the exacerbation frequency in the previous year, although treatment effects tended to be greater with higher number of exacerbations in the year prior to study entry. In another post hoc analysis of the above study, dupilumab (200 mg SC) every two or every four weeks was associated with clinically meaningful improvements in asthma control (as assessed by ACQ-5) and quality of life (assessed by AQLQ), while it also improved asthma symptoms and reduced productivity loss [128]. In another study of similar design, 1902 patients with severe uncontrolled asthma were assigned to receive dupilumab (200 or 300 mg SC) or matched placebo every two weeks for 52 weeks. The study again confirmed the favorable effect of both doses in reducing annual exacerbation rate and improving lung function. These effects, although observed irrespective of baseline blood eosinophils, were greater in those with > 300 cells/μL [129].

As with anti-IL-5 antibodies, dupilumab was assessed regarding its efficacy in reducing OCS in asthmatics with oral steroid-dependent asthma. Accordingly, 210 patients received dupilumab (300 mg) or placebo every two weeks for 24 weeks. Oral steroid doses were reduced from week four to week 20 and then remained at a stable dose for another four weeks. Dupilumab reduced oral corticosteroid dose by 70% compared to 42% reduction of placebo, and simultaneously decreased the rate of exacerbation by 59% compared to placebo; this effect was observed despite the reduction in the OCS dose. It also significantly improved lung function [130]. In the studies by Castro et al. and Rabe et al., transient eosinophilia was observed in few patients who received dupilumab.

A meta-analysis involving 3369 asthmatics from five studies concluded that treatment with dupilumab was effective in reducing exacerbations and improving lung function, asthma symptoms, asthma control and quality of life. Dupilumab was safe and well tolerated, and the most frequent adverse event was injection-site reaction [131].

Chronic rhinosinusitis with nasal polyps (CRSwNP) is often a comorbidity of severe eosinophilic asthma. In a subgroup analysis of a study involving patients with CRSwNP who received dupilumab as add-on therapy to mometasone fuorate nasal spray, those patients with comorbid asthma showed improvements not only in nasal polyp burden but also in asthma control, quality of life, and lung function [132].

## 8. Anti-IgE Therapy in Severe Asthma

### Omalizumab

Omalizumab is a humanized monoclonal antibody that binds free IgE and prevents it from binding to the high-affinity IgE receptor on basophils and mast cells [133]. Omalizumab is now approved for the treatment of moderate-to-severe allergic asthma in patients > 6 years of age. Omalizumab was the first biologic approved for use in asthma 15 years ago. To be eligible for omalizumab, the asthmatic should demonstrate sensitization to one of the perennial allergens on skin prick testing. Levels of total IgE combined with body weight are used to calculate the dose and the frequency of dosing. Omalizumab is administered subcutaneously either once a month or every two weeks. It was extensively studied in both clinical trials and real-world observational studies and was found to reduce the annual relative risk of asthma exacerbation by 38% and the risk of emergency visits by 47% compared with controls, according to pooled data from seven randomized studies [134]. The benefit of omalizumab in reducing exacerbations in relation to the presence of biomarkers reflective of T2 inflammation was evaluated in a study, showing that asthmatics with peripheral blood eosinophils ≥ 260 cells/μL and FeNO ≥19.5 ppb had a greater reduction of exacerbations compared to those with biomarker values below the above cut-off levels [135]. Accordingly, these biomarkers could be beneficial in selecting patients who are more likely to respond to omalizumab treatment. However, more recent data from real-world studies suggest that blood eosinophil levels are not predictors of reduction in exacerbations [136,137].

## 9. Other Therapies

Thymic stromal lymphopoietin (TSLP) is produced by airway epithelial cells in response to inhaled allergens and proinflammatory stressors [138,139].

Tezepelumab is a human monoclonal antibody that binds to TSLP, inhibiting its stimulatory action on dendritic cells and innate lymphoid cells, thus preventing the induction of type 2 cytokines (e.g., IL-5, IL-4, and IL-13). One phase II, randomized, double-blind, placebo-controlled trial evaluated the efficacy and safety of tezepelumab in patients with uncontrolled asthma, despite treatment with long-acting beta-agonists and medium-to-high doses of inhaled corticosteroids. Three dose levels of subcutaneous tezepelumab were compared to placebo over 52 weeks. The primary end point was the annualized rate of asthma exacerbations. Exacerbation rates were significantly reduced in tezepelumab groups—regardless of the baseline blood eosinophil count—compared to placebo by 61% in the low-dose group, 71% in the medium-dose group, and 66% in the high-dose group. Lung function was improved irrespective of the dose, while health-related quality of life improved only in the high-dose group [140].

Prostaglandin D2 (PGD2) is mainly released from mast cells, but platelets, alveolar macrophages, Th2 cells, and dendritic cells can also produce smaller amounts of PGD2. Prostaglandin D2 contributes to T2 inflammation through binding of the G-protein-coupled receptor chemoattractant receptor-homologous molecule expressed on Th2 cells (CRTH2) [141]. Fevipiprant is an oral competitive antagonist of CRTH2.

In a phase II study, including 170 patients with mild-to-moderate persistent, allergic asthma, fevipiprant produced a significant improvement in FEV1 AUC_0–24_ only in patients with high serum IgE and blood eosinophils > 300/µL [142].

In another phase II study, including 61 patients with moderate-to-severe, persistent asthma and sputum eosinophilia (≥ 2%), fevipiprant produced a significant, 3.5-fold greater decrease in sputum eosinophilia than placebo during the 12-week treatment period. In addition, fevipiprant reduced bronchial submucosal eosinophil numbers in bronchial biopsies compared to placebo. However, no change in blood eosinophil count was observed [143].

Finally, in another phase IIb study, including 1058 patients with allergic asthma uncontrolled with inhaled corticosteroids, fevipiprant—as well as montelukast—improved pre-dose FEV1 compared to placebo. However, no evidence of a higher efficacy in any predefined subgroup, including blood eosinophil count was observed [144].

## 10. Summary

In order to consider a biologic therapy for severe asthma, it is fundamental to firstly confirm asthma diagnosis and then solve possible problems related to non-adherence to medication, improper inhaler technique, and treatment of comorbid conditions.

For severe eosinophilic asthma, targeted therapies directed against IL-5 and IL-4 are available up to date (Table 1). These agents proved effective mainly in reducing asthma exacerbations but also in improving lung function and asthma control. It is clinically desirable that these antibodies seem to work specifically for uncontrolled asthma despite the use of daily oral corticosteroids. This led to the option of systemic steroids as the last alternative for GINA step 5, which will likely be entirely erased as a treatment option in the years to come.

Another major benefit from the use of biologics in severe asthma is the opportunity for a better insight into asthma pathophysiology mechanisms. An important but still unanswered question is whether biologics have an effect on moderate asthma or produce a disease-modifying effect. Until then, and while expecting more biologics to come (e.g., tezepelumab), we hope to gain experience and understand more from the longer use of the current anti-T2 biologics. 

## Figures and Tables

**Table 1:** Studies on biologic therapies for severe eosinophilic asthma.

Study	Medication	Patients	Duration	Outcome
Pavord et al. [73] (DREAM study) Phase III	Mepolizumab	621	52 weeks	Reduced number of exacerbations
Ortega et al. [96] (MENSA study) Phase III	Mepolizumab	576	52 weeks	Reduced number of exacerbations and improved lung function (FEV1), asthma control (ACQ-5), and quality of life (AQLQ)
Bel et al. [97] (SIRIUS study) Phase III	Mepolizumab	135	20 weeks	Reduced oral corticosteroid dose and number of exacerbations
Chapman et al. [102] (OSMO study) Phase III	Mepolizumab	145	32 weeks	Reduced number of exacerbations and improvement in asthma control (ACQ-5) and quality of life (SGRQ)
Chupp et al. [103] (MUSCA study) Phase III	Mepolizumab	551	24 weeks	Improvement in the SGRQ total score
Castro et al. [109] Phase III	Reslizumab	953	52 weeks	Reduced number of exacerbations and improvement in lung function (FEV1), asthma control (ACQ-7), and quality of life (AQLQ)
Bleecker et al. [114] (SIROCCO study) Phase III	Benralizumab	1205	48 weeks	Reduced number of exacerbations, improved lung function (FEV1), and asthma control
FitzGerald et al. [115] (CALIMA study) Phase III	Benralizumab	1306	56 weeks	Reduced number of exacerbations and improved lung function (FEV1)
Nair et al. [118] (ZONDA study) Phase III	Benralizumab	220	28 weeks	Reduced oral corticosteroid dose and number of exacerbations
Busse et al. [119] (BORA study) Phase III	Benralizumab	1576	56 weeks	Validated 2-year safety of benralizumab use
Wenzel et al. [127] Phase IIb	Dupilumab	769	24 weeks	Reduced number of exacerbations and improved lung function (FEV1)
Castro et al. [129] Phase IIb	Dupilumab	1902	52 weeks	Reduced number of exacerbations and improved lung function (FEV1)
Rabe et al. [130] Phase III	Dupilumab	210	24 weeks	Reduced oral corticosteroid dose, number of exacerbations, and improved lung function (FEV1)
Corren et al. [140] Phase II	Tezepelumab	550	52 weeks	Improved lung function (FEV1) and reduced number of exacerbations
Erpenbeck et al. [142] Phase II	Fevipiprant	170	28 days	Improved lung function (FEV1) in patients with high blood eosinophil number or high serum immunoglobulin E (IgE)
Gonem et al. [143] Phase II	Fevipiprant	61	12 weeks	Reduced sputum eosinophilia
Bateman et al. [144] Phase IIb	Fevipiprant	1058	12 weeks	Improved lung function (FEV1)

**Abbreviations:** FEV1—forced expiratory volume in the first second; ACQ—asthma control questionnaire; AQLQ—asthma quality of life questionnaire; SGRQ—Saint George’s respiratory questionnaire.
